# De la microscopía a la metagenómica: evolución y retos de la microbiología clínica

**DOI:** 10.7705/biomedica.8421

**Published:** 2026-04-28

**Authors:** Marylin Hidalgo

**Affiliations:** 1 Departamento de Microbiología, Pontificia Universidad Javeriana, Bogotá, D.C., Colombia Pontificia Universidad Javeriana Pontificia Universidad Javeriana Bogotá, D.C. Colombia

«¡Eureka!», podría uno imaginarse que exclamó Anton van Leeuwenhoek al ver por primera vez los microorganismos -protozoos y bacterias- a través de los microscopios diseñados por él mismo. Desde entonces, el microscopio se convirtió en la herramienta más utilizada en la microbiología clínica. Este, junto con la coloración de Gram, diseñada por Hans Christian Gram, han hecho de la microbiología un mundo fascinante.

A finales del siglo XIX, surgió la disciplina de la microbiología clínica, impulsada por el descubrimiento de los microorganismos como agentes causantes de enfermedades infecciosas y por la formulación de los postulados de Robert Koch.

El cultivo, es decir, el aislamiento del microorganismo continúa siendo la prueba de referencia para la microbiología, ya que nos permite tener acceso al microorganismo y practicar pruebas fenotípicas, genotípicas y de sensibilidad antimicrobiana. Este proceso hace posible identificar el microorganismo, lo cual le permite al médico clínico formular el tratamiento adecuado; sin embargo, este proceso puede tardar entre 24 y 72 horas, dependiendo del microorganismo, lo cual representa un factor limitante ante una situación crítica.

El uso de equipos automatizados para detectar microorganismos y establecer su sensibilidad a los antibióticos ha aportado un gran valor a los laboratorios de microbiología clínica, ya que minimiza los errores en la identificación del agente patógeno y reducen el tiempo para producir un resultado. Por otro lado, los equipos traen incluidas las pruebas de sensibilidad y generan un antibiograma adecuado, siguiendo las normas establecidas para tal fin. En la práctica, estos sistemas tienen una característica común: hacen el trabajo más amable, más eficiente y con menos errores por parte del operador.

Otra herramienta utilizada en la microbiología clínica es la prueba ELISA (*Enzyme-Linked Immunosorbent Assays*) (1960) que ha evolucionado hasta los formatos actuales en placas de microtitulación. Esta técnica ofrece gran sensibilidad y especificidad, aunque presenta limitaciones de tiempo, de disponibilidad de equipos y, además, no permite aislar el microorganismo, sino solamente observar la respuesta inmunológica del paciente. Existen también las pruebas de inmunoensayo de flujo lateral, las cuales son muy útiles, rápidas, de bajo costo y de gran utilidad en aquellas instituciones con recursos limitados.

Como respuesta a las limitaciones de los métodos tradicionales, particularmente a las del cultivo, se empezaron a utilizar los métodos moleculares, como la reacción en cadena de la polimerasa (PCR). Esta técnica le ofrece al laboratorio una gran ventaja: la detección rápida de microorganismos comunes y de difícil cultivo; además, su detección se puede obtener en un tiempo menor -incluso en horas-.

La PCR con todas sus variaciones -múltiple, microarreglos, de tiempo real y digital- le ofrece al laboratorio una opción rápida para detectar bacterias, parásitos, virus y hongos. Las casas comerciales han desarrollado variados estuches que le ayudan al laboratorio clínico, ya que el proceso de detección es automatizado y estandarizado, y puede dar resultados confiables. Sin embargo, las técnicas moleculares requieren equipos y reactivos especializados que están disponibles solo en los grandes centros médicos.

En los últimos años, la proteómica, particularmente mediante la MALDI- TOF MS (*Matrix Assisted Laser Desorption/Ionization - Time of Flight Mass Spectrometry*), se ha incorporado de forma rutinaria en muchos laboratorios de microbiología clínica. Esta herramienta permite identificar rápidamente, mediante la huella de masas peptídicas, bacterias, hongos y algunos péptidos virales, lo cual reduce con frecuencia el tiempo de diagnóstico en varias horas. Sin embargo, requiere un equipo especializado, el cual no pueden adquirir todos los centros de diagnóstico.

Por otro lado, la detección molecular ha avanzado hasta la secuenciación completa del genoma -*Whole Genome Sequencing* (WGS)-, lo cual ha transformado, en gran medida, la aproximación al agente patógeno, pues cuenta con múltiples plataformas de secuenciación y de ensamblaje que permiten conocer la secuencia completa del microorganismo. Esto es de gran utilidad para la detección temprana de brotes, y el monitoreo global de agentes patógenos emergentes y de interés en salud pública.

Recientemente, la secuenciación metagenómica de nueva generación (mNGS) (*Metagenomic Next-Generation Sequencing*), también conocida como metagenómica de tipo *shotgun*, permite detectar los agentes patógenos directamente a partir de las muestras clínicas, sin necesidad de cultivo. Con este enfoque, los ácidos nucleicos que son extraídos de una muestra -sangre, líquido cefalorraquídeo o secreciones respiratorias- se secuencian de manera aleatoria, generando información del genoma del huésped y de los microorganismos presentes. Posteriormente, se identifican las secuencias microbianas por medio del análisis bioinformático, lo cual es de gran utilidad para identificar el agente patógeno en infecciones de origen desconocido, como meningitis, fiebres y neumonías, entre otras.

Este es un panorama muy interesante, ya que nos permite ver la evolución de técnicas, herramientas y equipos para la detección de los agentes responsables de los cuadros clínicos de los pacientes.

Sin embargo, se debe tener en cuenta que es el resultado de un equipo, sin el conocimiento de la clínica del paciente. Este punto hace que surja la incógnita de si se está creando una brecha entre las máquinas y los bacteriólogos, los microbiólogos y los analistas de laboratorio. En este sentido, la capacitación continua del personal científico y del técnico es fundamental para comprender la lógica de la automatización y las bases biológicas de los procedimientos diagnósticos.

El laboratorio clínico enfrenta múltiples retos: el incremento de infecciones intrahospitalarias y extrahospitalarias, la emergencia de agentes patógenos zoonóticos y el rápido avance de la tecnología para detectar los agentes patógenos. Esta situación exige la rápida implementación de estas metodologías y la actualización constante del profesional del laboratorio en áreas nuevas como la bioinformática. También, constituye un reto para la academia, que no siempre avanza al ritmo vertiginoso de la tecnología en el laboratorio clínico.

Al final, más allá de la tecnología disponible, el verdadero valor del laboratorio clínico sigue estando en la capacidad del profesional para interpretar los resultados con criterio, integrarlos a la clínica y convertirlos en decisiones que impacten realmente la salud del paciente.
